# Riparian vegetation structure and the hunting behavior of adult estuarine crocodiles

**DOI:** 10.1371/journal.pone.0184804

**Published:** 2017-10-11

**Authors:** Luke J. Evans, Andrew B. Davies, Benoit Goossens, Gregory P. Asner

**Affiliations:** 1 Danau Girang Field Centre, Kota Kinabalu, Sabah, Malaysia; 2 Sabah Wildlife Department, Kota Kinabalu, Sabah, Malaysia; 3 Cardiff School of Biosciences, Cardiff University, Cardiff, United Kingdom; 4 Department of Global Ecology, Carnegie Institution for Science, Stanford, California, United States of America; 5 Sustainable Places Research Institute, Cardiff University, Cardiff, United Kingdom; Auburn University, UNITED STATES

## Abstract

Riparian ecosystems are amongst the most biodiverse tropical habitats. They are important, and essential, ecological corridors, linking remnant forest fragments. In this study, we hypothesised that crocodile’s actively select nocturnal resting locations based on increased macaque predation potential. We examined the importance of riparian vegetation structure in the maintenance of crocodile hunting behaviours. Using airborne Light Detection and Ranging (LiDAR) and GPS telemetry on animal movement, we identified the repeated use of nocturnal resting sites by adult estuarine crocodiles (*Crocodylus porosus*) throughout the fragmented Lower Kinabatangan Wildlife Sanctuary in Sabah, Malaysia. Crocodile resting locations were found to resemble, in terms of habitat characteristics, the sleeping sites of long-tailed macaque; positioned in an attempt to avoid predation by terrestrial predators. We found individual crocodiles were actively selecting overhanging vegetation and that the protrusion of trees from the tree line was key to site selection by crocodiles, as well as influencing both the presence and group size of sleeping macaques. Although these findings are correlational, they have broad management implications, with the suggestion that riparian corridor maintenance and quality can have implications beyond that of terrestrial fauna. We further place our findings in the context of the wider ecosystem and the maintenance of trophic interactions, and discuss how future habitat management has the potential to mitigate human-wildlife conflict.

## Introduction

Riparian areas often contain an above average level of biodiversity [1], and the use of riparian corridors is designed to provide contiguous access of isolated forest fragments to a range of species, thus preserving gene flow and preventing inbreeding [2], particularly for larger, wide-ranging vertebrates [3]. Apex predators are particularly sensitive to changes in prey base arising from the presence or absence of riparian corridors. Thus, the illegal destruction of, or failure to maintain, riparian zones during forest conversion, has the potential to disrupt trophic interactions and result in extinction debts that may take long periods to fully realise [4]. Sabah, Malaysian Borneo, is a large exporter of oil palm (*Elaeis guineensis*), which has driven widespread habitat conversion [5], including a large proportion of riverbank habitats. Despite this, and with the direct aim of preserving riparian corridors, local law prohibits planting of oil palm within 20 m of a major waterway (Sabah Water Resources Enactment 1998).

Despite high levels of deforestation, Sabah retains many large animal species, including the estuarine crocodile (*Crocodylus porosus*), the world’s largest reptile, reaching in excess of seven meters [6]. They are apex predators found throughout South and East Asia and into Australasia [7]. *Crocodylus porosus*, like most other crocodilians, are poikilothermic, basking diurnally as a means of regulating internal body temperature [8–9]. Crocodilians are primarily nocturnal hunters [9–10]; however, they appear to exhibit a similar nocturnal ‘basking’ behavior [11]. Whilst crocodilians are widely regarded as opportunistic feeders [12–13], their nocturnal hunting preferences are largely unknown. Moreover, the effects of habitat loss on these apex predators are yet to be quantified. The complex behaviors of many cryptic species, such as crocodilians, are understudied, with crocodilian research exploring little in the way of wild hunting behavior. Estuarine crocodiles are sit and wait predators, with identification of behavioristic routines playing a key role in hunting successes [14]. Sit and wait hunting strategy is particularly well represented within reptiles, such as a wide range of larger crocodilians [15], as well as snakes [16]. Conversely, many mammals utilize active hunting strategies, and even group hunting [17], to facilitate increased kill rates. Crocodilians ability to feed irregularly lends itself to this low energetic cost strategy.

Recent advances in remote sensing techniques, as well as GPS (Global Positioning System) tracking of cryptic species, have resulted in new insights into animal behavioral ecology [18–19]. These techniques have even extended to detecting individual behaviors on small temporal or spatial scales [20]. The combination of GPS tracking and high-resolution habitat mapping allows the detection of specific habitat requirements, as well as to produce informed management strategies [21]. Understanding how cryptic, apex predators utilize fragmented landscapes has implications for the management of the species, but in a broader sense, also the management of whole ecosystems as habitat. Crocodiles are sometimes referred to as a keystone species [22] and their ecosystem roles range from trophic management to ecosystem architecture [23]. Sabah is home to a stable population of estuarine crocodiles, and they are widely distributed throughout the state [24]. There is, however, evidence of falling crocodile numbers in rivers where increased levels of habitat loss have occurred (L. Evans, *unpubl*. *data*). It is likely that this is the result of prey reductions associated with habitat loss. The effect that this loss of prey accessibility has on crocodile feeding opportunities has not been quantified.

Primates form a significant portion of adult *C*. *porosus* diet, with widespread observations of feeding events (L. Evans, *pers*. *obs*.). The long-tailed macaque (*Macaca fascicularis*) is found throughout Sabah and, being widespread, is often regarded as a pest species. Macaques do, however, provide important sources of protein for a wide range of species including crocodiles [25–26], clouded leopards (*Neofelis diardi*) [27–28], reticulated pythons (*Python reticulatus*) [29], and even humans [30]. Long-tailed macaques often sleep in trees bordering large rivers [31–33], with one possible explanation, the predator avoidance hypothesis [31,34], ascribing this behavior to macaques avoiding predation by clouded leopards or reticulated pythons [29]. Such behavior results in a reduction in the area needing to be monitored for predators [29]. Increased protection from terrestrial predators may, however, make macaques vulnerable to crocodiles that can wait below the sleeping macaques in case they fall from the trees to escape other predators, or by simply losing their balance. Whether crocodiles do indeed make use of such a hunting strategy remains untested.

By combining high-resolution airborne LiDAR and GPS telemetry on animal movement, we aimed to determine estuarine crocodile use of riparian vegetation during hunting, and to assess and quantify the relationship between crocodile resting sites and hunting behaviors. We hypothesised that adult crocodilians were actively selecting nocturnal resting sites based on potential feeding opportunities. By examining the resting habitat choices of both crocodiles and long-tailed macaques, we sought to assess the importance of riparian vegetation in maintaining cryptic hunting behaviors.

## Methods

**Fig 1:**
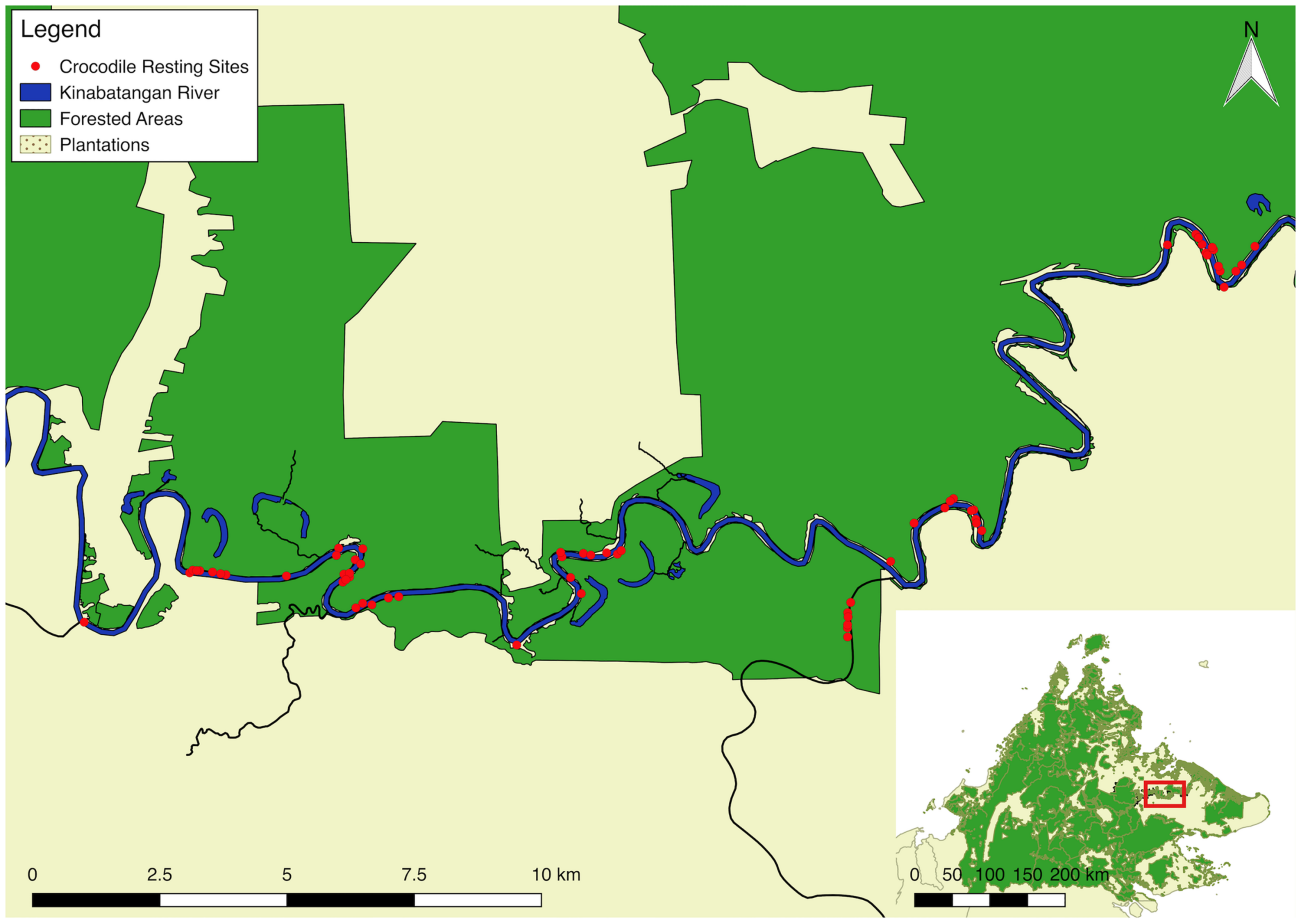
Nocturnal crocodile resting sites. Locations of crocodile resting sites determined through tLoCoH analysis, throughout the fragmented study site. Inset, the study sites location within Sabah.

The Lower Kinabatangan Wildlife Sanctuary (LKWS) (N5.403712, E117.998169) is a highly fragmented landscape comprised of degraded, logged forest and oil palm monoculture. The LKWS borders the largest river in Sabah and the second largest river in Borneo, the Kinabatangan. The river has a large flood basin with a catchment of approximately 16,800 km^2^ or around 23% of Sabah’s total land area [35], underscoring the importance of the ecosystems associated with this river. The LKWS is separated into ten distinct forested lots with varying levels of connectivity. In addition, there are a number of small forestry reserves (Class VI), under different jurisdiction. The study site comprised a ~50 km stretch of the Kinabatangan, as well as a number of tributaries and oxbow lakes (Fig 1). The area included lots 5–7 as well as the Pin Supu Forest Reserve. The study area was chosen due to a large population of estuarine crocodiles, as well as, its logistical favorability.

### Crocodile tagging

Estuarine crocodiles were captured using 3.66 m steel mesh traps with weight-loaded door pins triggered by bait manipulation. Traps were fitted with a series of fuel canisters to create buoyancy and allow the trap to float semi-submerged. Traps were set in areas of low current but deep water, with slip knots securing the trap to nearby vegetation, allowing for variations in the river’s water level.

GPS units were obtained from two different companies, African Wildlife Tracking (AWT)(Pretoria, South Africa) and E-Obs GMBH (Grünwald, Germany). These units operated differently, with AWT units allowing for two-way data transfer and E-Obs requiring manual ultra-high frequency (UHF) download. The error ranges of these two types of tags were, however, comparable (~20 m) and thus for the purpose of this study were treated as one. Units were fitted to the nuchal plate of the individuals in accordance with Kay [36], with the addition of sub dermal attachment [37].

### Macaque surveys

Nocturnal surveys to determine the presence of macaques in trees overhanging the river were conducted by boat by a team of two observers. Surveys were carried out between 19:30 and 23:00 local time (GMT+8) and encompassed both the main river and tributaries. Field surveys were carried out to account for changes in water level, which may have affected the tree protrusion score. Tree characteristics were collected regardless of macaque presence. Each tree was assessed for sleeping macaques, and, when detected, group size was estimated in intervals of five individuals. Their relative vertical position within the canopy (low-high) was also noted. In addition, each tree was assessed, and an approximate height (5 m increments) and protrusion index (1–5 scale) was estimated. The protrusion index was estimated based on the amount by which the tree was overhanging the river with an index value of 1 being outer leaves reaching the water’s edge and 5 indicating that the majority of the canopy was over the water. Finally, the presence, or absence, of crocodiles beneath each tree was recorded.

### Airborne LiDAR

We mapped the study area with discrete-return airborne Light Detection and Ranging (LiDAR) in April 2016 using the Carnegie Airborne Observatory-3 (CAO [38]). The CAO LiDAR subsystem provides three-dimensional structural information of vegetation canopies and the underlying terrain. The Global Positioning System-Inertial Measurement Unit (GPS-IMU) subsystem provides three-dimensional position and orientation data for the CAO sensors, allowing for highly precise and accurate positioning of LiDAR observations on the ground. For this study, the CAO data were collected from 3000 m above ground level, using a scan angle of 36° and a side overlap of 30%. The aircraft velocity was 150 knots and the LiDAR pulse frequency was set to 150 kHz, with a beam divergence of 0.25 mRad, resulting in an average point density of 3.20 laser shots per m^2^. Horizontal and vertical error estimates were 16 cm and 7 cm RMSE, respectively.

Laser ranges from the LiDAR were combined with the embedded GPS-IMU data [39] to determine the 3-D locations of laser returns, producing a ‘cloud’ of LiDAR data. The LiDAR data cloud consists of a large number of geo-referenced three-dimensional coordinates, where elevation is relative to a reference ellipsoid. Initially, the LiDAR data points were processed to identify which laser pulses penetrated the canopy volume and reached the ground surface using the ‘lasground’ tool packaged in the LAStools software package (Rapidlasso, Gilching, Germany). We used these points to interpolate a raster digital terrain model (DTM) for the ground surface. A second digital surface model (DSM) was based on interpolations of all first-return points (i.e. it includes canopy top and, where only ground returns exist, bare ground). Measurement of the vertical difference between the DTM and DSM yields a digital canopy model (DCM) throughout the study site.

The final LiDAR data products of ground elevation and woody canopy height were derived at 2 m spatial resolution. Although small changes in vegetation structure could have occurred during the six-month period between the end of the crocodile spatial and LiDAR data collection, we assumed that because we were only interested in large perennial trees, their general structure would remain largely unchanged over the course of the study period.

### Statistical analysis

Mean sizes and GPS tagging durations are presented as ± standard deviation, unless otherwise stated. GPS location data were analysed per individual crocodile and resting sites were identified using R (version 3.3.0) package tLoCoH (version 1.40.01). Local Convex Hull (LoCoH) analyses were tested and α LoCoHs deemed most suitable for the dataset. Data were limited to nocturnal hours, 1900–0600 (GMT+8), to control for hunting and ensure that resting sites were not being utilized for poikilothermic basking behavior. Figures of α were determined on a case-wise basis and ascertained by examining both the hulls respect for true boundaries and the inclusion of known gaps within the dataset, for example inaccessible areas. In order to avoid pseudo-replication, an inter-visit gap (IVG) of 48 hours was implemented. This was chosen due to the largely sedentary lifestyle of crocodilians and ensured that reutilisation of sites was a true response. Resting sites were selected from the generated dataset by using filters of both duration (>4 hours) and revisitation (>2 discrete occurrences separated by seven days). These parameters ensured that each location selected as a resting site was utilized for a minimum of eight hours.

Resting sites were identified using QGIS (version 2.18.0). Aggregated points within the perceived margin of GPS error were summarised using a 20 m buffer centralizing the aggregation. Vegetation lower than 3 m was masked out for ease of viewing and to remove shrub-like vegetation. Overhanging vegetation was determined by identifying a tree within the buffered sites that extended a minimum of 5 m from the tree line. Depending on water levels, this overhang could have represented either water or mud banks. In addition to the resting sites calculated by t-LoCoH, an equal number of random sites, within the study site, were selected and assessed for overhanging vegetation. Random sites, of equal number, were selected using the creation of a 1 m buffer along the edge of the riverbank and random locations within that buffer selected using QGIS. General habitat composition directly bordering river banks, namely, the presence of a corridor, with a corridor defined as riverbank forest presence greater than 20 m but less than 100 m in diameter, forest blocks, or oil palm plantations, was tested against resting site occurrence using linear regression. Overhanging vegetation proximity to resting sites and randomly selected sites was examined using a two sample T-test. Differences in vegetation characteristics between utilized and random sites were assessed using a binomial Generalised Linear Models (GLM). In addition, Poisson GLMs were constructed to assess the factors affecting utilization frequency of resting sites (Table 1). Model selection using corrected Akaike’s Information Criterion (AICc) values was utilized, through R package MuMIn (v. 1.15.6), to ensure the most parsimonious model (ΔAICc was > 2 between the selected models and gthe next best one) was selected [40]. For both GLMs, factors examined were tree height (th)(maximum tree height found within the buffer), total canopy area of the tree (ca), and protrusion from tree line (dt), as well as the two-way interaction between these factors. A binomial GLM was also constructed to examine the factors that affected the presence or absence of macaques during the field surveys. In this instance, tree protrusion and size of tree canopy (scored on a 1 (low) to 5 (high) scale) were examined. A two-sample T-tests was performed to analyze the effect of higher levels of protrusion on group size. Values of p<0.05 were utilized to denote significance in all non-AICc informed tests.

**Table 1:** Candidate models used to assess differences in vegetation between resting and random sites (Binomial GLMs) and factors affecting utilization frequency of resting sites (Poisson GLMs). Model selection was implemented using ΔAICc, and Akaike weights in the MuMIn R package for both model types. dt = protrusion from tree line; ca = total canopy area of the tree; th = tree height.

Model	AICc	ΔAICc	Akaike weight
***Binomial***			
**dt**	27.9	0	0.603
**dt+ca**	30.33	2.43	0.179
**dt+ca+ca:dt**	30.73	2.83	0.147
**dt+th+ca+ca:dt**	32.56	4.65	0.059
**dt+th+ca+th:dt+ca:dt**	35.72	7.82	0.012
***Poisson***			
**dt**	148.5	0	0.573
**dt+ca**	150.8	2.29	0.182
**dt+th**	150.8	2.3	0.181
**dt+ca+th**	152.9	4.37	0.064

## Results

A total of seven *C*. *porosus* were included in the study, with a mean body length of 3.94 m (± 0.64 m). Of the seven individuals tagged, five were male (4.08 m (± 63.46 cm) and two were female (3.54 m (± 58.69 cm). The capture of a larger number of males than females is consistent with the findings of other studies [41–42]. Individuals were tagged for a mean of 78.14 (± 65.25) days. Three additional tagged individuals did not provide sufficient data to be included in the study.

Across all tagged individuals, 169 discrete locations, spread across 46.4 km of main river habitat, satisfied the minimum duration and frequency of visitation parameters for nocturnal resting sites. A total of 163 of these locations were restricted to the main river, with an additional six being located in a major tributary. The majority of the locations were found along bank habitats bordering forest, although for 34 of these locations (20.86%), the forest fragments bordering the river were less than the legally required minimum of 20 m for riparian buffers, and in some cases were not present at all (Table 2). Differences in resting site occurrences per kilometre of riparian habitat exhibited a trend, but was found to be not significant (p = 0.063), likely owing to a lack of oil palm habitat within the study site. A further six locations were found to be within the river, outside of shallow hunting areas, and excluded from the study. Locations had a 20 m buffer added to compensate for GPS error and to aid in assessing vegetation overlap. This yielded 54 distinct locations for analysis with a mean utilization of 2.64 (± 2.53) visits, spread throughout the study area. The most heavily utilized location yielded 26 separate visits.

**Table 2:** Summary of resting site occurrences by habitat type.

River Bank Habitat	Total Riparian Habitat Prevalence (km)	Total Resting Site Occurrences	Resting Sites/km
Forest	50.19	127	2.53
Corridor (>20 m)	27.91	34	1.22
Oil Palm Plantation	6.26	2	0.32

**Fig 2:**
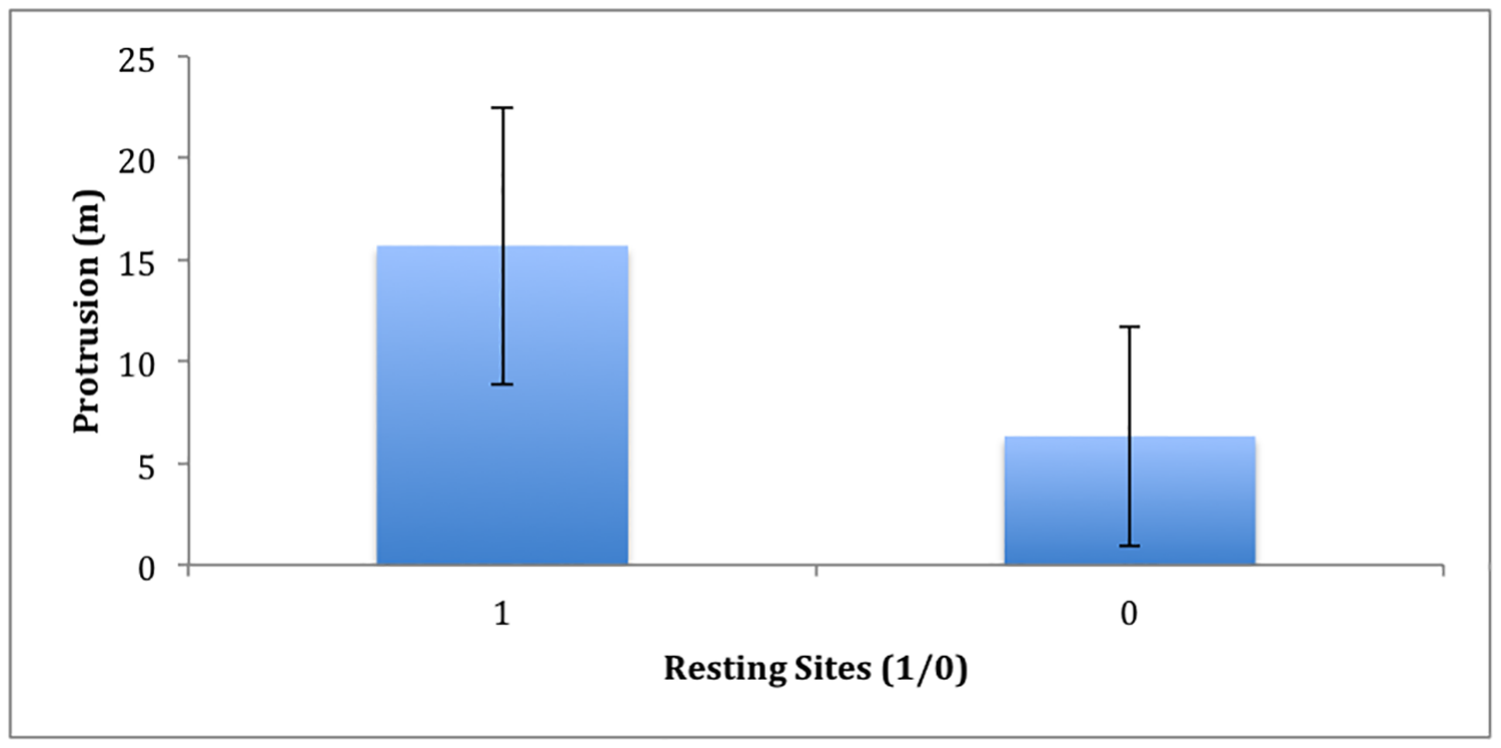
Protrusion of riverine trees from tree line. Protrusion levels for both crocodile resting (1) and randomly selected sites (0).

A total of 54 (31.95%) of the 169 resting sites identified were found to closely align with overhanging, protruding trees. In contrast, randomly selected sites were found to align with overhanging vegetation in just 25 (14.81%) of 169 occurrences. These figures represented a significant difference between utilized and random sites (t = 153.2463, df = 336, p<0.001). Tree height, tree canopy surface area, nor the interaction between these factors had a significant effect on either resting site selection or site utilization (p>0.05) (Table 1). However, the protrusion of the tree from the tree line was highly significant in the selection of resting sites (β = 5.99; p = <0.001) (Fig 2). Additionally, there was a significant correlation between resting site utilization and tree protrusion (β = 0.17; p = <0.01).

**Fig 3:**
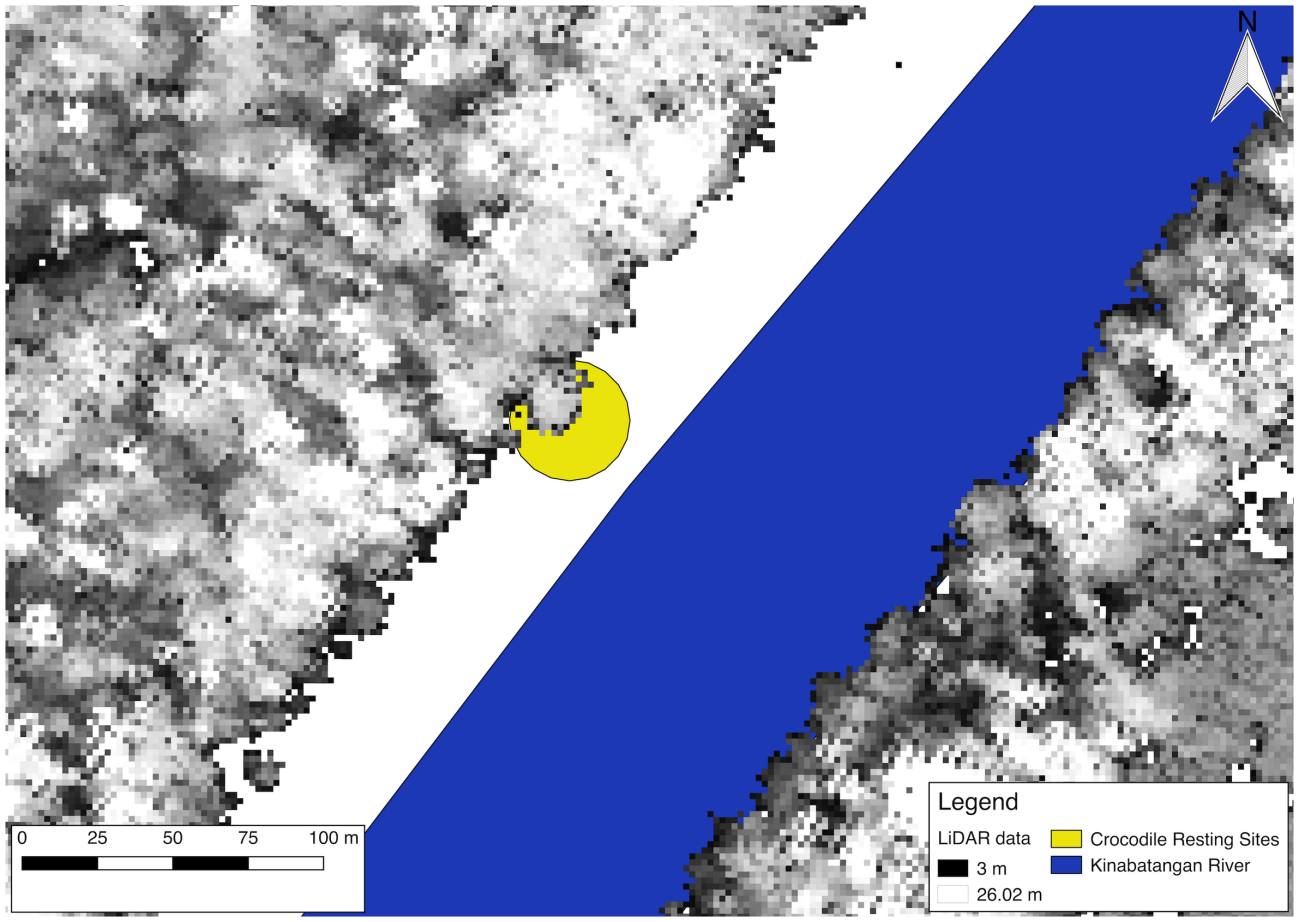
An example of a resting site and overhanging vegetation. Location derived from airborne LiDAR, associated with the location. This was also the most heavily utilized resting site with 26 separate visitations.

The most utilized location (n = 13) was found to align with a very large tree with a high protrusion index (Fig 3). Given the number of reutilizations and the parameters determining a resting site, this location was utilized by an individual (Male 5) for a minimum of 104 nocturnal hours over the course of 33 tagged nights.

A total of 229 trees were surveyed for sleeping macaques, with a recorded occupancy of 29.7%. Macaque groups averaged 8.47 (± 6.31) for confirmed individuals and 12.51 (± 6.31) for estimated group size. The mean protrusion index (1 = low– 5 = high) was 2.88 (± 1.44) for trees containing macaques and 2.28 (± 1.52) for those without, with a significant correlation between higher levels of tree protrusion and macaque presence (β = 0.75; p = 0.035). Macaques were also found in larger numbers (β = 0.016; p = <0.01) on trees that protruded further. They were also located at a variety of canopy heights with larger groups often spread throughout the vertical canopy, including within 1 m of the water surface. In addition, during the course of the surveys, two crocodiles were sighted underneath trees containing sleeping macaques, whilst no crocodiles were observed underneath trees without macaques.

## Discussion

Our results demonstrate correlational similarities in habitat requirements of both crocodiles and sleeping primates. These similarities suggest active crocodile hunting behavior, and this behavior’s reliance on the presence of protruding, or overhanging, trees. Large trees found in riverine habitats are among those felled first during deforestation, due to ease of access, however our results highlight the importance of high quality, riparian habitat for maintaining potential for access to prey sources and the importance of conservation buffer zones along rivers. These are essential for maintaining ecosystem functionality, especially in fragmented landscapes. Associated with this are important conservation planning implications.

Crocodiles were found to heavily reutilize sites with a high level of tree protrusion, showing preferences for particular locations, with up to 26 utilizations of a single site being recorded. An explanation for such behavior is that the site represents a fruitful opportunistic hunting location for the individual. In turn, this suggests that terrestrial habitat characteristics have the potential to influence the population stability of this aquatic reptile. Moreover, the quality of the remnant habitat appears to be of the greatest importance, with protruding trees providing potentially important hunting locations. The very high prevalence of long-tailed macaques found in these locations (29.7% of trees) ensures that given the longevity of the resting periods, a large degree of temporal overlap between the species would have occurred across the sites.

The presence of nocturnal resting sites is not surprising, given that crocodiles are most likely to prefer to rest in shallows rather than expend energy swimming against the current. The repeated use of discrete locations does, however, suggest an external factor is likely to be influencing the choice of resting sites among individuals. Given that crocodiles hunt primarily at night [41], and are ambush predators [12,43], these resting sites that are occupied for at least four hour periods over the course of a night are likely to be associated with hunting behavior. The site selection, in terms of tree protrusion index, as well as spatial overlap between the species, reinforces the notion that the crocodile’s active resting site selection is being influenced by the macaques’ choice of sleeping tree. Furthermore, increased levels of protrusion are likely to result in a higher proportion of successful feeding events, once an individual has fallen from the perch.

Insights into hunting behaviors of apex predators are notoriously difficult to observe, and thus the nuances of wild crocodilian hunting behaviors, as well as numerous others, are understudied. These insights are of particular importance in changing landscapes where ecosystem management is key to ensuring viable populations, especially given that through the conservation of top predators, other trophic levels can receive a net ecological benefit [44]. Through the use of GPS telemetry and LiDAR technologies, remote sensing of both individuals and habitat, LiDAR data have allowed for accurate analysis of tree metrics at the local scale, whilst providing an overview of riparian corridor width and quality throughout the study site and giving an insight into cryptic behaviors. Terrestrial habitat impacts on crocodilian behaviors are largely unknown, although previous studies have investigated terrestrial hunting prevalence [45], and climbing in juvenile crocodilians [11]. This study provides a first quantitative look at the importance of the interface between aquatic and terrestrial environments, with implications for future managements of tropical ecosystems.

Riparian corridors found throughout the study site tended to lack the large trees found directly in bank habitats surrounded by forest (L. Evans, *pers*. *obs*.), likely due to the increased access by plantation workers to the resources. Given the importance placed on protruding trees by both large reptiles and primates, the study highlights the importance of not just the maintenance of riparian corridors, but also the quality of habitat that remains, following adjacent land conversion. Fig trees (*Ficus* sp.), a group that represents the vast majority of overhanging trees in the LKWS, are particularly at risk of removal during riparian corridor loss, they represent important providers of fruit to a wide array of species found in the Kinabatangan, including macaques [46]. A range of riparian corridor widths exists throughout the study site and findings here provide added impetus to restore corridors, both in terms of quality and extent.

The importance of maintaining ready access to prey in areas containing large numbers of adult crocodilians could be viewed as purely self-serving exercise, with declining prey numbers likely to cause rises in human-crocodile conflict, especially in those areas that have high quantities of anthropogenic activity. Anthropogenic expansion brings its own threats to crocodile population stability; with increases in cases of human attacks often drawing calls for government-mediated culls or for personal revenge killings. Responses of this nature to wild animal populations have the potential to harm eco-tourism revenues, an important revenue stream for Sabah. The maintenance of primate prey sources has the potential aid in both the mitigation of human-crocodile conflict, particularly in areas containing large numbers of crocodiles, as well as the continued growth of the eco-tourism sector.

A potential limitation of our study was the amount of time between GPS (ranging from July 2011 –November 2015) and LiDAR (April 2016) data collections. Typically, however, long tree lifespans should have ensured that changes in large-scale vegetation structure were limited, with major discrepancies occurring only when trees fell into the river. As these forms of differences in the data would have produced false negatives, rather than false positives, the proposed arguments are, if anything, strengthened. Whilst the number of individual crocodiles utilized in this study was limited (n = 7), they represent the majority of territorially-active individuals in the study area, and are some of the largest individuals found within the study site. It is these individuals that are most likely to hunt larger vertebrates such as primates. Given that only tree protrusion was found to be significant, this study justifies the use of more readily available, cheaper satellite imagery, for future studies.

The status of the LKWS as a fragmented mosaic of large-scale agriculture and degraded tropical rainforest represents a model for the future status of many tropical ecosystems worldwide. This study will form part of an on-going effort to provide an updated management plan for estuarine crocodiles in Sabah. Additionally, the results should be used to provide leverage for further protection of riparian buffers throughout not just Sabah, but the entire species range. The importance of understanding species behavior in such ecosystems is paramount for ensuring continued ecosystem functionality throughout the tropics, and for conservation planning in both currently protected and unprotected areas.

## Supporting information

S1 File Tree habitat characteristics. LiDAR derived tree locations and habitat characteristics.
(CSV)

S2 File Macaque survey data. Habitat characteristics and group sizes determined during primate surveys.
(CSV)
